# Early Diagnosis and Vaginal Hysterectomy in Cancer of the Uterus*Read before the Tri-State Medical Society of Alabama, Georgia, and Tennessee, Chattanooga.

**Published:** 1897-04

**Authors:** J. A. Goggans

**Affiliations:** Alexander City, Ala., Grand Senior Counsellor of the Medical Association of the State of Alabama; Fellow of the Southern Surgical and Gynecological Society, Fellow of the British Gynecological Society


ATLANTA

### Medical and Surgical Journal.

Vol. XIV. APRIL, 1897. No. 2.

LUTHER B. GRANDY, M.D., MANAGING EDITOR.

M. B. HUTCHINS, M.D., BUSINESS MANAGER.

COLLABORATORS:
A. W. CALHOUN, M.D., LL.D., VIRGIL O. HARDON, M.D., FLOYD W. McRAE, M.D.,
A. W. STIRLING, M.D., JOSEPH EVE ALLEN, M.D., Augusta, Ga., and
HENRY R. SLACK, Ph.M., M.D., LaGrange, Ga.

### ORIGINAL COMMUNICATIONS.

### EARLY DIAGNOSIS AND VAGINAL HYSTERECTOMY IN CANCER OF THE UTERUS.*

By J. A. GOGGANS, M.D., Alexander City, Ala.,

Grand Senior Counsellor of the Medical Association of the State of Alabama; Fellow of the Southern Surgical and Gynecological Society, Fellow of the British Gynecological Society.

The problem of the pathogenesis of cancer is certainly one of the most interesting and important that is engaging the attention of the medical profession of the present day. While it is true that pathogenesis is not yet a settled question, certain results have been arrived at from clinical observation, and I feel that in presenting for your consideration the subject of this paper, viz.: "The Early Diagnosis and Vaginal Hysterectomy in Cancer of the Uterus," it is a very important one. I feel that it is a subject a knowledge of which the surgeon should be especially active in

*Read before the Tri-State Medical Society of Alabama, Georgia, and Tennessee, Chattanooga.

disseminating and in encouraging the general practitioner to investigate.

That the mortality from cancer within the past few years has been materially reduced should be generally known, and is due to three principal factors, viz.: (1) prophylaxis; (2) early recognition of the disease; (3) perfection in technique of all capital operations. The prophylaxis of cancer of the uterus consists in making a thorough examination of all women who have borne children, repairing all lacerations of the cervix, and correcting, as far as possible, all pathological conditions that may be present.

The early diagnosis of cancer of the uterus is not always an easy thing; but the happiness and life of many of our women depend upon our ability to early recognize the disease when present. Age, race, heredity and unsanitary environment are predisposing causes of cancer of the uterus. The following table given by Gusserow is very instructive:

AGE AT WHICH CANCEROUS DISEASE DEVELOPED IN 3,385 CASES.

| | |
|---|---|
| At 17 years | 1 case. |
| 19 years | 1 case. |
| 20 to 30 years | 114 cases. |
| 30 to 40 years | 770 cases. |
| 40 to 50 years | 1,196 cases. |
| 50 to 60 years | 856 cases. |
| 60 to 70 years | 340 cases. |
| Above 70 years | 193 cases. |

It will be seen that the largest number of cases of cancer of the uterus is developed between the ages of forty and fifty years, and by carefully noticing the age limits of this table, it will be found of considerable help in the early recognition of cancer.

The onset of the disease is usually so insidious that the general health may be remarkably preserved, while the disease may be far advanced. The first symptom that the patient notices is hemorrhage, which may be only slight at first, but gradually becomes more profuse on the slightest exertion, and many assume some periodicity, or may even become continuous. To this is soon added pain. This, however, may not be excessive until the whole uterus and surrounding tissues become involved. Leucorrhea may be a prominent symptom, and the whole train of reflex nervous phenomena from the disordered digestive tract, and the circulation, complete the rational symptoms. On making a vaginal examination the cervix will be found to be indurated, and may be nodular, but the mobility of the uterus is not yet diminished. An ocular examination will reveal a somewhat elevated surface around the os which bleeds from the slightest touch. This surface gradually ulcerates and becomes yellowish on the surface. In most cases these symptoms are sufficient for a diagnosis, but should doubt still exist as to the true nature of the disease, sections of the deep tissues of the cervix should be made and submitted to a microscopic examination.

In the cases on which I have operated, a microscopic examination was not absolutely necessary, the disease having been already so far advanced that the diagnosis was easily made.

An examination of the statistics from 1877 down to the present time will reveal a gradual but enormous decline in the percentage of mortality of vaginal hysterectomy for cancer of the uterus. Brunner, in 1884, collected all the cases then published, before 1877, 33 in number, with a mortality of 82 per cent. From that time down to 1887 the mortality decreased to 24.47 per cent.:

The following table given by Martin, 1886, is exceedingly interesting and gives an average mortality of only 15 per cent.

| | |
|---|---|
| Fritsch, 60 operations | 7 deaths. |
| Leopold, 42 operations | 4 deaths. |
| Olshausen, 47 operations | 12 deaths. |
| Schröder and Hoffmeier, 74 operations | 12 deaths. |
| Staude, 22 operations | 1 death. |
| A. Martin, 66 operations | 11 deaths. |
| Average | 15 per cent. |

But this is not the climax as the following will show: The latest statistics give as an average mortality about 5 per cent. These include series of cases reported by Leopold, 80 in number, with 4 deaths, 5 per cent. Kattenbach, 53 cases and 2 deaths, 4 per cent. D. de Ott, 30 cases, and Pean, 25 cases, each without a death. The low mortality obtained by Dr. Jacobs, of Brussels, as well as many operators in this country, deserves to be mentioned as giving only about 4 per cent. mortality. These figures seem to me to be sufficient to place vaginal hysterectomy for cancer among the most legitimate of operations and should encourage a genuine reaction against all partial extirpations of the uterus. Consequently (I shall not attempt to describe the different operations for cancer of the cervix not involving the vaginal vaults and the fundus) *I adhere to the idea that if the lesion is cancerous, no matter how small, the operation of vaginal hysterectomy should be performed.*

It should be remembered that the main object to be attained is the ability to make an early diagnosis, for it is only by operating early that the disease can be totally eradicated.

I shall not attempt to give the technique of the operation of vaginal hysterectomy, but will content myself by stating that I prefer the method of first opening Douglas's pouch by free incision, and hemostatic suture of the pelvic floor, and finally displacement of the uterus backward and ligation of the broad ligaments. The after treatment is very simple. The iodoform gauze drain should be removed about the fourth or fifth day; but vaginal irrigations should not be employed until seventh or eighth day. The sutures should not be removed until the patient is able to leave her bed, which is usually at about the end of the third week. The general management of these cases is about the same as after any ordinary abdominal operation.

My object in bringing this subject before this society is not to produce anything new, but merely to elicit discussions on a subject that I fear is too little appreciated by general practitioners of medicine. Up to within the past few years the subjects of uterine cancer were considered incurable, and were advised to take opiates to subdue pain until death could relieve them. The improved technique of vaginal hysterectomy now offers much encouragement for this class of sufferers. Indeed no achievement of this age could add more pleasure to the human race than the discovery of the exact pathogenesis, and a complete and successful treatment for cancer.

---

The doctor sees all the weakness of mankind, the lawyer all the wickedness, the theologian all the stupidity.—*Schopenhauer.*

